# Determination of Acr-mediated immunosuppression in *Pseudomonas aeruginosa*

**DOI:** 10.1016/j.mex.2022.101941

**Published:** 2022-11-28

**Authors:** Benoit J. Pons, Edze R. Westra, Stineke van Houte

**Affiliations:** Environment and Sustainability Institute, Biosciences, University of Exeter, Penryn, UK

**Keywords:** CRISPR-Cas, Anti-CRISPR, Transformation efficiency

## Abstract

Bacteria have a broad array of defence mechanisms to fight bacteria-specific viruses (bacteriophages, phages) and other invading mobile genetic elements. Among those mechanisms, the ‘CRISPR-Cas’ (Clustered Regularly Interspaced Short Palindromic Repeats – CRISPR-associated) system keeps record of previous infections to prevent re-infection and thus provides acquired immunity. However, phages are not defenceless against CRISPR-based bacterial immunity. Indeed, they can escape CRISPR systems by encoding one or several anti-CRISPR (Acr) proteins. Acr proteins are among the earliest proteins produced upon phage infection, as they need to quickly inhibit CRISPR-Cas system before it can destroy phage genetic material. As a result, Acrs do not perfectly protect phage from the CRISPR-Cas system, and infection often fails. However, even if the infection fails, Acr can induce a lasting inactivation of the CRISPR-Cas system. The method presented here aims to assess the lasting CRISPR-Cas inhibition in *Pseudomonas aeruginosa* induced by Acr proteins by:

• Infecting the *P. aeruginosa* strain with a phage carrying an *acr* gene.
• Making the cell electrocompetent while eliminating the phage
• Transforming the cells with a plasmid targeted by the CRISPR-Cas system and a non-targeted one to measure the relative transformation efficiency of the plasmids.

This method can be adapted to measure which parameters influence Acr-induced immunosuppression in different culture conditions.

## Graphical abstract

**Figure:**
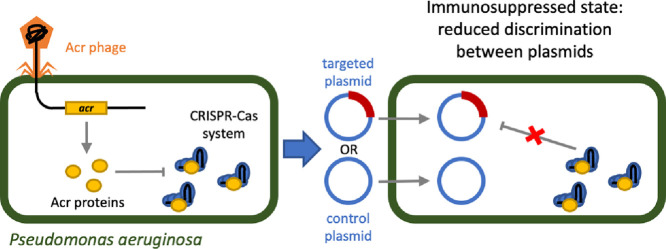

Specifications table

**Table:** 

Subject area	Biochemistry, Genetics and Molecular Biology
More specific subject area	Microbiology, phage biology
Name of your method	Determination of Acr-mediated immunosuppression in *Pseudomonas aeruginosa*.
Name and reference of original method	M. Landsberger, S. Gandon, S. Meaden, C. Rollie, A. Chevallereau, H. Chabas, A. Buckling, E. R. Westra, S. van Houte, Anti-CRISPR Phages Cooperate to Overcome CRISPR-Cas Immunity, Cell 174 (2018) 908-916. doi:10.1016/j.cell.2018.05.058
Resource availability	*P. aeruginosa* UCBPP-PA14 - O'Toole Lab (NCBI: NC_008463.1) *P. aeruginosa* UCBPP-PA14 *csy3::LacZ* - O'Toole Lab *P. aeruginosa*SMC4386 – Davidson Lab (NCBI: NZ_LOQZ00000000.1) DMS3*mvir* - O'Toole Lab DMS3*mvir*-AcrIF1 - Bondy-Denomy Lab DMS3*mvir*-AcrIF4 - Bondy-Denomy Lab pHERD30T plasmid - Davidson lab (GenBank: EU603326.1)

## Method details

### Introduction

The CRISPR-Cas (Clustered Regularly Interspaced Short Palindromic Repeats – CRISPR-associated) system is a bacterial defence system used to prevent invasion by Mobile Genetic Elements (MGEs), including bacteriophage (phages) [1]. CRISPR-Cas systems are classified in 2 classes, 6 types and 33 subtypes [2]. CRISPR-Cas mode of action relies on a three-step process (reviewed in [3]). First of all, fragments (protospacers) from invading MGEs are inserted in one of the CRISPR arrays on the bacterial chromosome. Then, protospacers are expressed as CRISPR RNAs (crRNAs) and associate with Cas proteins to form a so-called ‘surveillance complex’. Finally, during subsequent invasion from the same MGE, the surveillance complex will be able to recognise, bind and cleave the invading genetic material. The protospacers have a fixed length and can only be acquired from the MGE or bound by the surveillance complex if they are flanked by a Protospacer Adjacent Motif (PAM) [4]. Protospacer length and PAM sequences are specific to each CRISPR-Cas subtype.

However, phages are not defenceless against CRISPR-Cas targeting. They can carry anti-CRISPR genes (*acr*), which lead to the production of Acr proteins early upon infection [5]. Acr need to be produced in a timely manner to prevent the CRISPR-Cas system from cleaving the phage genetic material [6]. Therefore, Acr sometimes fails to protect phage from the CRISPR-Cas system [7]. However, they induce a lasting immunosuppression state [7,8], which this protocol aims to measure.

This method relies on transformation of a targeted plasmid, carrying a protospacer recognised by the CRISPR-Cas system of interest, and a control non-targeted plasmid, without protospacer. We describe here the use of pHERD30T plasmid, a P_BAD_-based shuttle vector carrying a gentamicin resistance marker [9]. This plasmid was selected because it is a well characterised *P. aeruginosa* plasmid and it carries a resistance gene for gentamicin, to which *P. aeruginosa* PA14 is sensitive. The protocol can be adapted to other *P. aeruginosa* plasmids with a suitable resistance marker. The method was originally developed to measure immunosuppression induced by AcrIF1 and AcrIF4 on type I-F CRISPR-Cas system carried by *Pseudomonas aeruginosa* UCBPP-PA14 (PA14). We also describe how to adapt to other CRISPR-Cas system, by giving the example of type I-E CRISPR-Cas system carried by *P. aeruginosa* SMC4386.

## Strains and materials

a. Plasmid, bacteria, and phage strains

- pHERD30T plasmid.
- *Escherichia coli* DH5α chemically competent cells, either commercially available or homemade (efficiency ≥ 10^8^ cfu/µg DNA).
- Isogenic CRISPR immune and non-immune *Pseudomonas aeruginosa* strains (*e.g.*, PA14 *csy3::LacZ* SMC4386).
- Isogenic phages with and without anti-CRISPR genes (*e.g.*, DMS3m*vir*, DMS3m*vir*-AcrIF1, DMS3m*vir*-AcrIF4).

b. Molecular biology reagents and DNA oligonucleotides

- HindIII-HF, EcoRI-HF, CutSmart® buffer (NEB, USA).
- ATP (NEB, USA).
- T4 Polynucleotide Kinase (PNK), T4 PNK buffer (NEB, USA).
- T4 DNA ligase, T4 buffer (NEB, USA).
- Protospacer oligonucleotides (see design tips in section 3.a.).
- Sequencing oligonucleotides (*e.g.*, pHERDseq_for 5’-TCTCCAACCCGTTTTTTTGGGCTAGAA-3’, pHERDseq_rev 5’-CCGCGCTCGACTAACCCAG-3′).
- DNA clean-up kit (*e.g.*, Monarch® PCR & DNA Clean-up Kit (NEB, USA)).
- Plasmid miniprep kit (*e.g.*, GeneJET Plasmid Miniprep Kit (Thermo Fisher, USA)).
- Plasmid midiprep kit (*e.g.*, Qiagen Plasmid Midi kit (Qiagen, Germany)).

c. Bacterial culture media, reagents

- LB broth, LB agar (Thermo Fisher, USA).
- Agar (VWR, USA).
- 5-Bromo-4-chloro-3-indolyl-b-Dgalactopyranoside (X-Gal) (VWR, USA).
- Isopropyl β-D-1-thiogalactopyranoside (IPTG) (VWR, USA).
- Gentamycin sulfate (VWR, USA).
- Sucrose (Thermo Fisher, USA).
- Glycerol (VWR, USA).

d. Materials and consumables

- Heat block or water bath.
- (Optional) Programmable PCR thermocycler.
- UV spectrophotometer.
- Electroporator.
- 1.5 mL centrifugation tubes.
- Cell culture tubes.
- 250 mL and 500 mL glass flasks.
- 50 mL screw-cap tubes.
- 96-well plate.
- Electroporation cuvettes.

## Targeted plasmid construction

a. Protospacer design

In order to be recognised by the CRISPR-Cas system, the targeted plasmid needs to include a sequence matching one of the strain's spacers, flanked by the Protospacer Adjacent Motif (PAM). For the *Pseudomonas P. aeruginosa* PA14 strain, we used a protospacer matching spacer 1 of CRISPR array 2 of the type I-F CRISPR-Cas system [10]. The protospacer sequence was cloned in plasmid pHERD30T at the HindIII restriction site in the multiple cloning site. The sequence of interest in the plasmid was the following:

**Table:** 

HindIII restriction site	Spacer sequence	PAM	HindIII restriction site

A / AGCTT	ACCGCGCTCGACTACTACAACGTCCGGCTGA	GG	A / AGCTT

The oligonucleotides designed for cloning contain the protospacer sequence flanked by the PAM sequence, surrounded by overhangs of the HindIII restriction site (overhangs in small caps, protospacer in capital letters, PAM underlined):

- 5’-agcttACCGCGCTCGACTACTACAACGTCCGGCTGATGGa-3’
- 5’-agcttCCATCAGCCGGACGTTGTAGTAGTCGAGCGCGGTa-3’

When adapting this experiment to a new strain, we advise to design at least two different targeted plasmids, targeted by different spacers from different CRISPR arrays, and to test them to select plasmids with the lowest EOT (efficiency of transformation) on CRISPR immune bacteria (see “protospacer choice” section). Please note that sequence and position of the PAM depends on the CRISPR-Cas system type and that restriction sites used for cloning can be different than the one proposed here.

To adapt this method to *P. aeruginosa* SMC4386 type I-E CRISPR-Cas system, we selected and tested protospacers matching spacer 1 from the CRISPR array 1 and by spacers 1 and 7 from CRISPR array 2 (cr1sp1, cr2sp1 and cr2sp7, respectively). These protospacers were inserted between the HindIII and EcoRI restriction sites of pHERD30T multiple cloning site. The sequence of interest in the plasmid were the following:

**Table:** 

Spacer	HindIII restriction site	PAM	Spacer sequence	EcoRI restriction site

cr1sp1	A / AGCTT	AAG	AGAGTGAGCAGGTTCGTCCCCTTGGCACTCTG	G / AATTC
cr2sp1	A / AGCTT	AAG	AACCTCTACGAGCAGACCGAGTTGAAAGGGCA	G / AATTC
cr2sp7	A / AGCTT	AAG	GTGATGGAGCGGACCGCCCCGAGCACCGCAGA	G / AATTC

The oligonucleotides designed for cloning contain the protospacer sequence flanked by the PAM sequence, surrounded by overhangs of the restriction site (overhangs in small caps, protospacer in capital letters, PAM underlined):

- cr1sp1

∘ 5’-agcttAAGAGAGTGAGCAGGTTCGTCCCCTTGGCACTCTGg-3’
∘ 5’-aattcCAGAGTGCCAAGGGGACGAACCTGCTCACTCTCTTa-3’

- cr2sp1

∘ 5’-agcttAAGAACCTCTACGAGCAGACCGAGTTGAAAGGGCAg-3’
∘ 5’-aattcTGCCCTTTCAACTCGGTCTGCTCGTAGAGGTTCTTa-3’

- cr2sp7

∘ 5’-agcttAAGGTGATGGAGCGGACCGCCCCGAGCACCGCAGAg-3’
∘ 5’-aattcTCTGCGGTGCTCGGGGCGGTCCGCTCCATCACCTTa-3’

b. Targeted plasmid cloning
a. Vector preparation

- Set-up the following plasmid digestion reaction in a 1.5 mL microcentrifuge tube:

**Table:** 

Component	Concentration	Volume (µL)

pHERD30T	/	v
CutSmart buffer	10X	10
Ultrapure water	/	86 - v
HindIII-HF	20 units/µL	2
EcoRI-HF	20 units/µL	2
**Total volume**	**/**	**100**

*Add a volume v that provides 5 µg of plasmid

- Digest at 37 ˚C for 4h in a heat block or water bath.
- Purify the digested plasmid with PCR clean-up kit (*e.g.*, Monarch® PCR & DNA Clean-up Kit), according to the manufacturer's instructions; elute in 50 µL of ultrapure water.
b. Insert preparation
- Order or synthesise the oligonucleotides (see section 3.a for design tips).
- Resuspend primers in ultrapure water to reach a concentration of 100 µM (following the manufacturer's instructions).
- For each oligonucleotide pair, set-up the following phosphorylation reaction in a 1.5 mL microcentrifuge tube:

**Table:** 

Component	Concentration	Volume (µL)

Oligonucleotide 1	100 µM	2
Oligonucleotide 1	100 µM	2
ATP	10 mM	2.5
PNK buffer	10X	2.5
Ultrapure water	/	15.5
T4 Polynucleotide Kinase	10 units/µL	0.5
**Total volume**	**/**	**25**

- Run phosphorylation reaction at 37°C for 3h in a heat block or water bath.
- Heat-inactivate the phosphorylation reaction by transferring the microcentrifuge tube at 65 ˚C for 10 min in a heat block or water bath.
- Add 3 µL of CutSmart® buffer to the reaction tube and run the anneal program:

∘ *Option 1:* heat the microcentrifuge tube at 98°C for 5 min in a heat block; turn off the heat block and let the reaction mix cool down to room temperature.
∘ *Option 2:* transfer the reaction mix to a PCR tube and run the following cycle in a PCR thermocycler.

**Table:** 

Temperature	Duration

98°C	5 min
96°C to 22°C (-2°C/step)	30 s/step
20°C	hold

- Dilute 1 µL of the annealed oligonucleotides in 50 µL of ultrapure water in a 1.5 mL microcentrifuge tube
c. Ligation and transformation
- Set-up the following ligation reaction, using the undiluted digested pHERD30T (step 3.a) as vector and the 1:50 diluted annealed oligonucleotides (step 3.b.) as insert:

**Table:** 

Component	Concentration	Volume (µL)

Digested pHERD30T	Undiluted	1
Annealed oligonucleotides	1:50	1
T4 DNA ligase buffer	10X	1
Ultrapure water	/	6.5
T4 DNA ligase	400 units/µL	0.5
**Total volume**	**/**	**10**

- Run ligation at room temperature for 10 min
- Transform 5µL of ligation mix in commercially available chemically competent *E. coli* DH5α (high efficiency) as per manufacturer's instructions or in home-made chemically competent *E. coli* DH5α (efficiency ≥ 10^8^ cfu/µg DNA).
- Add 950 µL of LB to the transformation mix.
- Incubate at 37 ˚C for 1h at 180 rpm.
- Plate 50 and 500 µL on LB agar supplemented with 30 µg/mL of gentamicin sulfate, 200 µg/mL of X-Gal and 1 mM of IPTG.
- Incubate the plates at 37 ˚C for 12 to 18h.
- **Tip**: the transformation should yield a few hundred clones, with at least a few dozen white clones.

d. Cloning verification

- Select 5 to 10 individual white clones, pick them with a toothpick and add each clone to 5 mL of LB supplemented with 30 µg/mL of gentamicin sulfate in cell culture tubes.
- Incubate at 37 ˚C, 180 rpm for 12 to 18h.
- Purify the plasmids with plasmid DNA isolation kit (*e.g.*, GeneJET Plasmid Miniprep Kit), according to the manufacturer's instructions; elute in 50 µL of ultrapure water.
- Determine the concentration and purity of the purified plasmids using a UV spectrophotometer.
- **Tip**: Plasmid preps should yield around 200 ng/µL of DNA with an A260/A280 ratio of 1.8–2.0.
- Verify the correct insertion of the protospacer by sequencing the plasmids with pHERDseq_for and pHERDseq_rev oligonucleotides
- Select one of the plasmids carrying the correct protospacer

c. Protospacer choice (optional step)

- Prepare electrocompetent cells [11]:

∘ Prepare a culture of the chosen strain (*e.g., P. aeruginosa* PA14 or SMC4386) in fresh LB medium; incubate for 12-18 hours at 37°C, 180 rpm.
∘ **Tip**: the amount of overnight culture depends on the number of targeted plasmids to test; prepare 3 mL of overnight culture for the non-targeted plasmid and 3 mL of overnight culture per targeted plasmid to be tested. For the SMC4386 protospacer tests, we used 12 mL of overnight culture.
∘ Transfer overnight culture in 1.5 mL microcentrifuge tubes (2 tubes for the non-targeted plasmid and 2 tubes per targeted plasmid to be tested). For the SMC4386 protospacer tests, the culture was divided in 8 tubes.
∘ Centrifuge for 2 minutes at 16,000 g, discard the supernatant and resuspend the pellet of each tube in 1 mL of 300 mM sucrose at room-temperature.
∘ Repeat previous step.
∘ Centrifuge for 2 minutes at 16,000 g, discard the supernatant and resuspend the pellet of each tube in 50 µL of 300 mM sucrose at room-temperature.

- Transform cells with pHERD30T and the different targeted plasmids variants (see step 3.b for targeted plasmid production):

∘ Mix 100 µL of electrocompetent cells with 100 ng of plasmid in a 1 mm wide electroporation cuvette
∘ Insert the cuvette in the electroporator and electroporate at 25 µF, 200 Ω, 2.5 kV (**Tip**: the time constant displayed after electroporation should be inferior to 5 ms)
∘ Add 900 µL of fresh LB medium to the electroporated cells and transfer to a 2 mL microcentrifuge tube; incubate cells for 1 hour at 37°C, 180 rpm.
∘ Centrifuge for 2 minutes at 16,000 g, discard the supernatant and resuspend the pellet of each tube in 100 µL of fresh LB medium.
∘ In a 96-well plate filled with 180 µL of LB medium in each well, serial-dilute the resuspended cells from 10^−1^ to 10^−8^ by 20 µL transfer.
∘ Spot 20 µL of each dilution on LB agar supplemented with 50 µg/mL of gentamicin sulfate; incubate for 12-18 hours at 37°C.

- Count the number of transformants for each plasmid.
- Select the targeted plasmid that displays the lowest transformation efficiency compared to pHERD30T. This plasmid is then referred as pHERD30T-targ. For the SMC4386 protospacer tests, the selected plasmid was pHERD30T-cr2sp1 (Figure 1).

**1:**
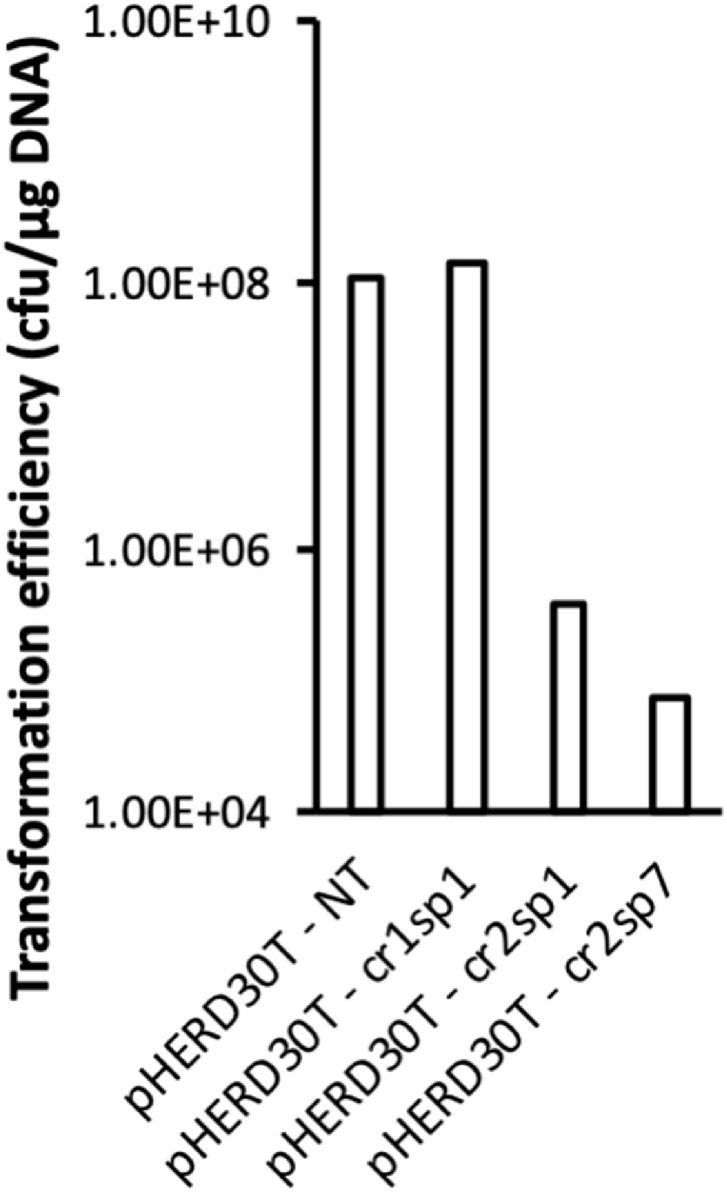
Protospacer selection for *P.* aeruginosa SMC4386 type I-E CRISPR-Cas system. Transformation efficiency of the pHERD30T plasmid non-targeted (NT) or carrying a protospacer matching the 1^st^ spacer from CRISPR array 1 (cr1sp1), the 1^st^ spacer from CRISPR array 2 (cr2sp1) or the 7^th^ spacer from CRISPR array 2 (cr2sp7).

d. Plasmid stock production

- Transform 50 ng of pHERD30T and pHERD30T-targ in commercially available chemically competent *E. coli* DH5α (subcloning efficiency) as per manufacturer's instructions.
- Add 950 µL of LB to the transformation mix, incubate at 37 ˚C for 1h at 180 rpm.
- Plate 50 µL on LB agar supplemented with 30 µg/mL of gentamicin sulfate
- Incubate the plates at 37 ˚C for 12 to 18h.
- Select one clone from each construction, add them to 5 mL of LB supplemented with 30 µg/mL of gentamicin sulfate in cell culture tubes.
- Incubate at 37 ˚C, 180 rpm for 12 to 18h.
- Transfer 500 µL of each overnight culture into 100 mL of LB supplemented with 30 µg/mL of gentamicin sulfate in a 500 mL glass flask.
- Purify the plasmids with plasmid midiprep DNA isolation kit (*e.g.*, Qiagen Plasmid Midi kit), according to the manufacturer's instructions.
- Determine the concentration and purity of the purified plasmids using a UV spectrophotometer. Plasmid preps should yield around 500 ng/µL of DNA with an A260/A280 ratio of 1.8–2.0.

## Infection

- Prepare cultures of the chosen *P. aeruginosa* strains, including a control strain without a functional CRISPR-Cas system (*e.g., P. aeruginosa* PA14 and PA14 *csy3::LacZ*) in fresh LB medium in a 250 mL glass flask; incubate for 12-18 hours at 37°C, 180 rpm.
- **Tip**: the amount of overnight culture needed depends on the number of phage treatments; prepare 20 mL plus 10 mL per phage treatment (*e.g.*, 50 mL for 3 phage treatment with DMS3*mvir*, DMS3*mvir*-AcrIF1 and DMS3*mvir-AcrIF4*). If the total volume exceeds 50 mL, use a 500 mL glass flask, or divide in two different 250 mL glass flask to allow sufficient oxygenation of the culture.
- Determine the cell density through OD_600_ measurement.
- **Tip**: for *P. aeruginosa* PA14, a 1:5 dilution of stationary phase culture should reach OD_600_ = 0.6, which represents a titre of 3.5×10^9^ Colony Forming Units (CFUs)/mL in the undiluted culture.
- Divide each culture in 10 mL fractions in 50 mL screw-cap tubes (1 tube per phage treatment, plus 1 tube for the ‘no phage’ control).
- Add phage in the ‘phage treatment’ tubes with a Multiplicity Of Infection (MOI) of 0.3 (*e.g.*, a final concentration of 1.1×10^9^ Plaque Forming Units (PFUs)/mL of DMS3*mvir* when infecting a stationary phase culture of *P. aeruginosa* PA14); incubate for 2 hours at 37°C, 180 rpm.
- Centrifuge the 50 mL screw-cap tubes at 3,500 rpm for 15 minutes at room temperature and discard the supernatant.
- **Optional step**: keep a 500 µL sample of supernatant from each 50 mL screw-cap tube for phage titration control.

## Transformation

- Resuspend the pellet of each 50 mL screw-cap tube in 1 mL of 300 mM sucrose at room-temperature, and transfer into 1.5 mL microcentrifuge tubes.
- Centrifuge for 2 minutes at 16,000 g at room temperature, and discard the supernatant.
- Resuspend the pellet of each tube in 1 mL of 300 mM sucrose at room-temperature.
- Centrifuge for 2 minutes at 16,000 g at room temperature, and discard the supernatant.
- Resuspend the pellet of each tube in 300 µL of 300 mM sucrose at room-temperature.
- **Optional step**: keep a 100 µL sample of electrocompetent cells from each tube for non-transformed cell titration control.
- Mix 100 µL of electrocompetent cells with 500 ng of pHERD30T and 100 µL of electrocompetent cells with 500 ng of pHERD30T-targ in two 1 mm wide electroporation cuvette.
- Electroporate at 25 µF, 200 Ω, 2.5 kV.
- Add 900 µL of fresh LB medium to the electroporated cells and transfer to 2 mL microcentrifuge tubes; incubate cells for 1 hour at 37°C, 180 rpm.
- Centrifuge for 2 minutes at 16,000 g, discard the supernatant and resuspend the pellet of each tube in 100 µL of fresh LB medium.

## Bacteria and phage controls

- In 96-well plates filled with 180 µL of LB medium in each well, serially dilute the transformed cells until 10^−7^ by 20 µL transfer.
- Spot 50 µL of each dilution on LB agar supplemented with 50 µg/mL of gentamicin sulfate.
- **Optional step**: phage control:

∘ In 96-well plates filled with 180 µL of M9 minimal medium in each well, serially dilute the supernatant samples from 10^−1^ to 10^−8^ by 20 µL transfer.
∘ Spot 5 µL of each dilution on a mix of molten LB soft agar (0.5%) with 5% (v/v) of an overnight culture of the phage titration strain (*e.g., P. aeruginosa* PA14 *csy3::LacZ* grown in LB) poured over LB agar.

- **Optional step**: non-transformed cells control:

∘ In 96-well plates filled with 180 µL of LB medium in each well, serially dilute the electrocompetent cells samples from 10^−1^ to 10^−8^ by 20 µL transfer.
∘ Spot 50 µL of each dilution on LB agar.

- Incubate all plates for 12-18 hours at 37°C
- Count the number of transformant of pHERD30T and pHER30T-targ for each tested condition. The Relative Transformation Efficiency (RTE) is calculated as RTE = number of pHER30T-targ transformants/number of pHERD30T transformants.
- **Optional step:** count the number of colonies and plaques on the bacteria and phage control plates to assess if the phage and bacteria concentrations are variable across the different tested conditions.

## Method validation

In Landsberger *et al.* [7] (figure 5), this experiment was performed using the strain *P. aeruginosa* PA14 (carrying a functional type I-F CRISPR-Cas system) and its lytic phage DMS3*mvir* (*a priori* targeted by PA14 CRISPR-Cas system). The RTE of the BIM2 strain (carrying 3 spacers against DMS3*mvir*) was close to 10^−4^, thus demonstrating efficient targeting of the plasmid, while the RTE of PA14 *csy3::LacZ* was close to 1 as the latter strain is not immune against pHERD30T and pHERD30T-targ transformation. The RTE of the BIM2 strain infected by DMS3*mvir-*AcrIF1 was significantly higher than the RTE of non-infected cells. Moreover, infection by a phage lacking AcrIF1 did not disturb the RTE of this strain and none of the phage infection disturbed the RTE of the control strain. Therefore, the observed RTE increase of the BIM2 strain infected by DMS3*mvir-*AcrIF1 is specific to the presence of both a functional CRISPR-Cas system in the host strain and an *acr* gene in the phage genome. This demonstrates that this protocol efficiently measures Acr-induced immunosuppression in *P. aeruginosa*.

## Ethics statements

This protocol does not include human or animal subjects.

## CRediT authorship contribution statement

**Benoit J. Pons:** Methodology, Validation, Writing – original draft, Visualization. **Edze R. Westra:** Supervision, Project administration, Funding acquisition, Writing – review & editing. **Stineke van Houte:** Supervision, Project administration, Funding acquisition, Writing – review & editing.

## Data Availability

Data will be made available on request.
